# Is It Time to Integrate Frailty Assessment in Onconephrology?

**DOI:** 10.3390/cancers15061674

**Published:** 2023-03-08

**Authors:** Henry H. L. Wu, Rajkumar Chinnadurai, Robert J. Walker, Karthik K. Tennankore

**Affiliations:** 1 Renal Research Laboratory, Kolling Institute of Medical Research, Royal North Shore Hospital, The University of Sydney, Sydney, NSW 2065, Australia; 2 Department of Renal Medicine, Northern Care Alliance NHS Foundation Trust, Salford M6 8HD, UK; 3 Department of Medicine, Dunedin Campus, University of Otago, Dunedin 9016, New Zealand; 4 Department of Medicine, Dalhousie University and Nova Scotia Health, Halifax, NS B3H 4R2, Canada

**Keywords:** older population, frailty, frailty assessment, onconephrology, chronic kidney disease, cancer

## Abstract

**Simple Summary:**

There are an increasing number of older people living with kidney cancer and/or cancer and kidney disease worldwide, sparking a wider discussion on the impact of frailty and the clinical significance of conducting frailty assessments for this patient population. We provide an update on the current evidence related to frailty assessment in onconephrology and identify areas where further research efforts are anticipated to address knowledge gaps within this topic.

**Abstract:**

Onconephrology has emerged as a novel sub-specialty of nephrology dedicated to the intersection between the kidney and cancer. This intersection is broad and includes a number of important areas of focus, including concurrent chronic kidney disease (CKD) and cancer, acute kidney complications of cancer, and cancer-treatment-induced nephrotoxicity. The importance of onconephrology is even more evident when considering the global growth in the population of older adults, many of whom are living with some degree of frailty. Furthermore, a considerable proportion of older adults have CKD (some of whom eventually progress to kidney failure) and are at high risk of developing solid tumour and hematologic malignancies. Specific to kidney disease, the association between frailty status and kidney disease has been explored in depth, and tools to capture frailty can be used to guide the management and prognostication of older adults living with kidney failure. Whilst there is emerging data regarding the assessment and impact of frailty in onconephrology, there remains a relative paucity of knowledge within this topic. In this article, we evaluate the definition and operationalization of frailty and discuss the significance of frailty within onconephrology. We review evidence on current approaches to assessing frailty in onconephrology and discuss potential developments and future directions regarding the utilization of frailty in this patient population. A greater awareness of the intersections and interactions between frailty and onconephrology and further efforts to integrate frailty assessment in onconephrology to optimize the delivery of realistic and goal-directed management strategies for patients is needed.

## 1. Introduction

Onconephrology has emerged as an intriguing new field in nephrology that addresses the complexity of care for patients suffering concurrently from cancer and kidney disease. The intersection between the kidneys and cancer is multi-directional, and our understanding of this relationship is rapidly changing through novel discoveries [1]. Broadly categorized, onconephrology encompasses individuals with kidney cancer, those living with pre-existing chronic kidney disease (CKD) and cancer (or with cancer prior to developing CKD), those living with cancer who develop acute complications related to the kidney (e.g., tumour lysis syndrome leading to acute kidney injury (AKI)), and those who sustain cancer-treatment-induced nephrotoxicities [1].

Frailty is an age-associated clinical syndrome characterized by a decreased ability to respond to stressor events and a state of vulnerability, as a consequence of cumulative physiological decline and accumulating deficits [2]. The prevalence of frailty amongst individuals aged ≥ 65 living with CKD is more than 10%, according to previous systematic review findings [3]. Frailty is independently linked with adverse clinical outcomes in all stages of CKD and has been repeatedly shown to be associated with increased risks of mortality and hospitalization in the CKD population [4,5,6]. Frailty status has also demonstrated significant associations with adverse outcomes in dialysis and transplantation, as well as AKI [6,7,8,9,10,11]. The decline from a state of being fit to frail is usually dependent on multiple factors, not limited to sarcopenia, chronic inflammation, impaired metabolism leading to acidosis, and cellular senescence, all of which are pathophysiological processes associated with CKD [12,13,14,15,16]. Other pathophysiological processes inherent to CKD are likely to further exacerbate this decline. Frailty has been invariably linked to cancer, with the complex pathophysiological processes associated with frailty and cancer playing an important role in exacerbating a cancer patient’s health status and subsequently their life expectancy [17,18,19]. An older, frailer cancer patient has reduced tolerance to cancer treatments such as chemotherapy and/or surgery and is at increased risk of treatment toxicity and complications [20,21].

While there has been a considerable focus over the last several years on ascertaining the relationship between frailty and kidney disease as well as frailty and cancer, there is little guidance on how frailty may impact onconephrology specifically and how best to utilize frailty assessments to manage older adults living with kidney cancer and its associated complications, as well as those living with concurrent cancer and kidney disease. In this article, we will review the definition and operationalization of frailty and discuss the scale and significance of frailty in onconephrology. We will evaluate currently available evidence on approaches to the assessment of frailty in onconephrology and conclude by providing a framework to develop and optimize the utilization of frailty in this nascent but rapidly developing field.

## 2. Definition and Operationalization of Frailty

With the increase in the aging population worldwide, the demographic of patients with kidney disease and, separately, those with underlying cancer, is changing. As evident from large national and international studies, there has been a pronounced increase in the number of older adults across the spectrum of kidney disease [22,23]. Similarly, some reports have projected that the incidence of cancer diagnoses among individuals 65 years and older may double from 6.7 million in 2012 to 14 million by 2035 [24]. However, while these changes may suggest an even worse prognosis for chronologically older adults with these conditions, this is not always the case [25]. This may partially be related to the fact that biological age does not equate to chronological age, and underlying conditions (including kidney disease) may accelerate biological aging [26,27]. This emphasizes the need to better capture those who are truly at risk of poor health outcomes, beyond chronological increases in age.

One way to better capture biological aging is by identifying individuals with impairments across a number of health domains, who may, in turn, be at increased vulnerability to poor health outcomes (irrespective of changes in chronological age). This accumulation of deficits leading to vulnerability is often termed as “frailty”. Importantly, in a modified Delphi process inclusive of geriatrics specialists, non-geriatrician physicians, other health professionals, and scientists, 107 statements regarding frailty were considered to help build a consensus definition [28]. Overall, 39 statements had a strong agreement for inclusion as part of the definition in the domains of “framework”, “frailty versus disability”, and “biomarkers” [28]. Some of the key constructs helping to define frailty included consideration that it is a dynamic process, increases vulnerability to poor health outcomes, involves alterations in multiple body systems, is not a disability, and can be reversed or attenuated. While this working definition is helpful, the operationalization of frailty is of even greater importance to clinicians, wishing to incorporate a measurement of frailty into clinical practice.

### 2.1. Kidney Disease

By far the tool that has the most application in the field of kidney disease is the Fried frailty phenotype (FFP). Initially developed by Fried and colleagues in 2001, the FFP is characterized as any three of slowness (based on walking speed), weakness (based on low grip strength), weight loss (based on a response to a question and/or change in weight), exhaustion (based also on a response to two question items), and low physical activity (using established questionnaires). Frailty, ascertained using the FFP, has been shown to associate with poor outcomes in CKD, dialysis, and post-transplantation [11,29,30]. However, while easy to interpret, the FFP lacks some construct validity. Many consider frailty to be a continuum, across which patients progress from a state of being clinically well to one of being more severely frail. Thus, a single binary measure (frail or not frail) may not fully capture small but important changes in frailty severity that is more characteristic of a chronic disease population.

In that regard, efforts have been made to quantify frailty using an approach based on deficit accumulation known as a frailty index (FI). FIs are disease-specific and are typically comprised of 30–40 variables (where a given condition is either present or absent) across multiple domains (function, comorbidity, mobility, social function, and cognition) [31]. Variables need to have characteristic properties; they must be associated with health status, their prevalence must increase with age, they cannot saturate too early, they must consider a range of systems, and the items must be the same if being evaluated serially on the same population. Although less well studied in kidney disease, higher severity of frailty using an FI approach is associated with morbidity and mortality in dialysis and for those waiting for a kidney transplant [32,33]. While more biologically representative of the construct of frailty, FIs may be cumbersome, especially if data to develop them are lacking or hard to acquire. Therefore, other tools that may be more pragmatic have been developed. The clinical frailty scale (CFS) is a categorical tool that rates individuals from a score of 1 “very fit” to 8 “very severely frail” and studies have shown that the CFS has value in outcome prediction as a more comprehensive tool [34,35,36]. However, while pragmatic tools based on clinical impression (such as the CFS) allow more efficiency and easier incorporation into clinical care, they are subjective. Interestingly, it has been shown that clinical impressions of frailty (either by patients or providers) do not always demonstrate a high level of agreement with objective measures [11,33,37,38].

Other frailty assessment tools rely on physical examination and the completion of tests to ascertain physical performance. The short physical performance battery (SPPB) is a test of standing balance, walking, and standing from a seated position. Based on the time a standing position is held, the time to complete the walking component, and the time to complete five chair stands, patients are assigned a score ranging from 0 to 12. Once again, the SPPB has been shown to have prognostic value [37,39]. Similarly, other physical examination tests (i.e., the sit-to-stand test) are also associated with morbidity and mortality in sub-populations of individuals with kidney disease [40].

A number of other tools have been developed to capture frailty status and severity [41]. While each has its own strengths and limitations, perhaps even more emphasis should be placed on using multiple tools that capture different aspects of frailty. It has been shown that there is limited overlap in the proportions of patients identified as being frail when measured using different tools, although each independently predicts poor health outcomes [33,37]. However, while the use of multiple tools may ensure a more global picture of frailty, it is limited by issues of pragmatism and practicality. Nonetheless, the broad implications of frailty for prognosis in the setting of kidney disease suggest that determining the best way to measure and utilize it as part of the standard of care is an area of high priority. Table 1 summarizes the characteristics of commonly used frailty measures in studies of kidney disease.

### 2.2. Oncology

Frailty has long been recognized as an area of critical importance to the oncology community, primarily to guide treatment decisions and provide an idea of long-term prognosis for patients with both hematologic and solid-tumour malignancies [42,43,44]. One of the original tools to measure functional status, the Karnofsky performance status (KPS) is an ordinal tool to assess how patients with underlying cancer can perform tasks, with scores ranging from 100 (normal, no complaints; no evidence of disease) to 10 (moribund; fatal processes) to deceased (score of 0) [45]. First created in 1948 for a population of patients receiving chemotherapy for lung disease, the KPS has been studied in a number of populations of individuals with cancer and has moderate concordance with clinician/patient-ascertained performance status [46]. Like the KPS, in 1960, the Eastern Cooperative Oncology Group (ECOG) derived a 6-point score of functional performance ranging from 0 (fully active) to 5 (deceased), with similar prognostic value [43].

Although both the KPS and ECOG have a long history of utilization in a number of populations, similar to the CFS, they rely on clinician impression, agreement between clinicians for either score is only in the moderate range at best, and patient and clinician scores have poor agreement [46,47]. Not surprisingly, objective measures to assess performance status have been studied (summarized by Scott et al.) [48]. While these measures are likely to see more use in clinical practice, some may have limited feasibility for widespread use due to the need for additional physical and person resources to complete them as part of existing care pathways. Table 1 also summarizes the characteristics of commonly used frailty measures in studies of oncology.

### 2.3. Cognitive Frailty

While much of the attention thus far has focused on physical performance, cognitive frailty is emerging as a concept that requires additional attention in kidney disease and oncology. Although variably defined, cognitive frailty is often considered the co-existence of cognitive impairment and physical frailty [49]. In studies of both kidney disease and cancer, cognitive impairment has been shown to have a direct impact on treatment (dialysis, transplant, and chemotherapy) outcomes and may underlie the reason not to pursue treatment. Although most frailty measures focus solely on physical frailty, the FI (being broadly aimed at capturing impairments in multiple health domains) often does include some metric of cognitive impairment, if included as one of the domains [31,33].

### 2.4. Geriatric Assessment

Another way to best identify all aspects of frailty (i.e., impairments across multiple health domains) is comprehensive geriatric assessment (CGA). CGAs are detailed assessments of overall health that incorporate formal assessments of cognition, mood, nutrition, functional status, and comorbidity, often utilizing multiple different information sources [50]. CGAs have been proven to be useful for prognostication in both populations; however, they require time and expertise [51,52,53]. Therefore, while useful to guide whether a given patient may be capable of receiving treatment, they may not be easily incorporated into a busy practice. Therefore, it seems reasonable to screen for frailty using a more simplistic tool (with high sensitivity) and use the CGA for those with high frailty severity where knowing the determinants of frailty may impact treatment decisions.

### 2.5. Assessing Frailty Using a Virtual Approach

One of the primary limitations with the assessment of frailty is the importance of assessing patients face to face, most importantly, when physical examination may be required. Although this is a barrier to capturing frailty (that may be especially relevant given the changes in care that have paralleled the COVID-19 pandemic), it may not be insurmountable. In both kidney disease populations and in those with underlying malignancy, virtual assessments of frailty may be potential solutions [54,55]. These virtual solutions involve a combination of questionnaire-based assessment tools and video assessments to capture physical examination elements of a frailty assessment (i.e., gait speed), with or without a caregiver for those who are severely frail. While the literature surrounding virtual assessments of frailty is emerging and these approaches require validation, they may be novel ways to overcome limitations with face-to-face approaches.

## 3. Clinical Significance of Frailty Assessment in Onconephrology

It is recognized that kidney cancer, specifically RCC, affects older adults more significantly than younger individuals [56]. To date, older patients have been under-represented in clinical trials evaluating RCC outcomes and treatment efficacy [57,58]. The extent to which older patients with RCC are affected by the underlying condition and treatment is likely greater than what has been currently concluded [58]. Many older adults are burdened with age-associated kidney function decline (estimated to be at an average estimated glomerular filtration rate (eGFR) reduction of 8 mL/min/1.73 m^2^/decade) and increased number of co-morbidities (including hypertension, atherosclerosis, and diabetes mellitus), this being even more so for patients living with concurrent cancer due to a reduction in homeostatic resilience and physiological reserve from aging [59,60]. It is inevitable, therefore, that many patients with RCC will succumb to developing CKD, especially those who lose functioning nephron mass through partial or complete nephrectomy.

The increased risk of cancer amongst older CKD patients is well considered, despite these links not being as fully established. Linear associations between the risk of cancer and declining eGFR (that accompanies increasing age) have previously been demonstrated, particularly in advanced CKD and end-stage kidney disease [61,62,63]. Older patients with cancer are more susceptible to acute kidney complications, where there is an increased incidence of critical care admissions and uptake of kidney replacement therapy [64]. This population is also at greater risk of sustaining cancer treatment-induced and other drug-induced nephrotoxicities, with increased co-morbidity status, age-associated eGFR decline, and poor compliance to treatment being significant risk factors alongside advancing age [58,65,66].

Therefore, the basis of pursuing frailty assessment in different settings of onconephrology could be justified from two major dimensions. Firstly, the prevalence of kidney cancer, concurrent cancer with CKD, AKI with cancer, and treatment-associated nephrotoxicities in cancer patients are significantly greater amongst older patients [67,68,69]. Older patients with cancer tend to have greater levels of frailty in comparison to individuals without cancer [17]. Furthermore, data (albeit limited) suggest that patients with kidney cancer, concurrent cancer with CKD, AKI with cancer, and/or treatment-associated nephrotoxicities most likely have worsened clinical outcomes with increased frailty [66,70,71,72,73]. Considering these factors, frailty status would be useful as a marker to guide clinical decision-making in onconephrology.

### 3.1. Renal Cell Carcinoma

For patients with localized kidney cancer, frailty assessments may be helpful in guiding decisions surrounding the choice of nephrectomy procedure (i.e., radical vs. partial nephrectomy or radiofrequency ablation for smaller tumours) [74,75,76]. In most cases, it is well established that a radical nephrectomy may not be an appropriate option for older adults living with frailty, and nephron-sparing procedures (radiofrequency ablation) or partial nephrectomy are the treatment of choice for localized small RCCs depending on site of the lesion [74,75,77]. Frailty assessment may also be utilized to determine whether non-surgical treatment for localized kidney cancer may indeed be a better option. The use of ablative therapies such as cryotherapy and radiofrequency could be an attractive therapeutic option for older patients living with frailty avoiding by risks of operative morbidity. Previous systematic reviews evaluating the outcomes of older RCC patients receiving cryoablation versus undergoing a partial nephrectomy reported higher rates of tumour progression with cryoablation, but both treatment modalities had similarly low rates of distant metastases of <2% [78].

On the other hand, the prognosis would be expectedly poor among older adults with frailty and advanced RCC. It is estimated approximately 17% of older patients aged > 70 with metastatic RCC would be diagnosed with moderate or severe frailty, according to a retrospective study conducted by Pierantoni and colleagues [70]. In this study, there were significant differences in median progression-free survival (18.9 vs. 11.2 vs. 5.1 months, *p* < 0.001) as well as median overall survival (35.5 vs. 14.6 vs. 10.9 months, *p* < 0.001) between ‘fit’, ‘vulnerable’, and ‘frail’ older metastatic RCC patients. When compared to older ‘fit’ RCC patients, ‘frail’ patients had a lower chance of receiving second-line treatment if the first line was unsuccessful (28.9 vs. 66.6%, *p* = 0.002) in controlling RCC activity and advancement. The incidence of cancer treatment toxicities, in particular G3/4 toxicities, was significantly higher amongst ‘frail’ RCC patients compared to ‘fit’ patients (45 vs. 19%, *p* = 0.0025). In addition to providing an idea about prognosis, frailty assessments would be helpful to guide the choice and planning of treatment in metastatic kidney cancer. For immunotherapy, the CheckMate-214 and Keynote-426 trials, which included older patients but excluded those with frailty, suggested clinical and quality of life (QoL) benefits with a nivolumab–ipilimumab and pembrolizumab–axitinib combination in metastatic RCC [79,80,81]. However, perhaps counter-intuitively, if a major determinant of frailty severity in a patient with metastatic kidney cancer is attributed to their extensive cancer burden, these immunotherapy combinations may be the optimal option as treatment targets the leading factor [81]. However, older patients with cancer are often medically complex with multi-factorial contributors to their frailty severity which makes clinical decision-making regarding treatment more challenging [82]. Single-agent immunotherapy (e.g., single-agent pembrolizumab) has been advocated as a viable option for older patients with metastatic RCC. A phase 2 single-arm trial in the sub-study of Keynote-427 for previously untreated patients with advanced clear cell RCC demonstrated an objective response rate of 33.6% and a median progression-free survival of 6.9 months for patients receiving single-agent pembrolizumab [83]. Furthermore, 86% of patients had a positive response to treatment, with their responses lasting for at least 3 months [83]. These results suggest single-agent pembrolizumab possesses clinical benefits and a manageable toxicity profile for it to be considered in older patients with metastatic RCC [83]. There are increasing suggestions that anti-angiogenic therapy (i.e., VEGF tyrosine kinase inhibitors such as sunitinib and pazopanib) may present as a good alternative systemic therapy option to immunotherapy for older adults with frailty, but further validation regarding their clinical and QoL efficacy in this population is needed [84]. The role of cytoreductive nephrectomy, if a viable option, has also been considered in the context of an older, frail patient. Nevertheless, previous results have suggested older patients with frailty are much more likely to have higher peri-operative mortality if they were to undergo cytoreductive nephrectomy compared to younger patients, so at present, most would not be offered this treatment option [85].

Ultimately, frailty could be utilized in the setting of metastatic RCC to optimize care in those individuals who fail first-line chemotherapy. For example, if a patient with underlying frailty (especially if related to multiple factors) deteriorated following initial systemic therapy, subjecting them to additional treatments would not be expected to improve either quality or quantity of life. Outcomes from frailty assessments could be used to guide realistic discussions with patients, families, and multi-disciplinary teams on decision-making surrounding the futility of treatment, considering that continuous intervention may have significant potential in reducing patient QoL for very little gain in survival time [18,86,87]. Further study is required to determine how best to integrate frailty assessment for these purposes within the context of metastatic RCC.

### 3.2. Concurrent Cancer and Kidney Disease

Outside of kidney cancer, a frailty assessment may be utilized to prognosticate outcomes in older patients living with concurrent CKD and cancer as well as the risks of AKI and cancer-treatment-associated complications. In a retrospective study of 533 hospitalized patients aged ≥ 65 who had their creatinine levels measured at least more than once during admission for a period of 1 year, Baek and colleagues [64] identified 54 patients (10.1%) who developed AKI. The authors examined five variables (activity of daily living [ADL] and instrumental ADL dependence, dementia, nutrition, and polypharmacy) from a CGA and categorized patients into three groups according to the tertile of aggregate frailty scores: group 1, (score 1–2); group 2, (score 3–4); and group 3, (score 5–8). For older patients with cancer, it was concluded that the effects of frailty on AKI risk were particularly apparent compared to those without cancer (reference group 1, group 3: HR = 7.829, 95% CI 1.607–10.486, *p* = 0.003). The authors found that discriminatory accuracy for AKI incidence substantially improved with the addition of tertiles of aggregate frailty score to co-variates (AUC 0.641, AUC 0.739, *p* = 0.004). Therefore, frailty may be helpful for the prognostication of AKI, especially for those with concurrent cancer.

Recently presented at the 2023 American Society of Clinical Oncology Genitourinary Cancers Symposium was a multi-centre retrospective study that evaluated the impact of neoadjuvant-chemotherapy-induced AKI on oncological outcomes in patients who underwent radical cystectomy due to muscle-invasive bladder cancer (MIBC). Fujita and colleagues [88] noted that AKI during neoadjuvant chemotherapy is associated with worse oncologic outcomes in patients with MIBC. Amongst 398 patients with MIBC (median age of 69 years) who received 2 to 4 cycles of neoadjuvant chemotherapy followed by radical cystectomy, 66 patients (17%) developed AKI. The AKI group had a significantly lower proportion of patients with cancer staging less than ypT2 (38% vs. 53%) and downstaging (53% vs. 69%) compared with the non-AKI group. These findings understate the importance of considering frailty in onconephrology. Although frailty status was not evaluated in this study, it reaffirms that frailty may play a key role in AKI development and poor prognosis in cancer.

## 4. Current Approaches to Frailty Assessment in Onconephrology

Although a gold-standard frailty assessment framework has not yet been introduced, current approaches to detect and stratify frailty status in onconephrology aim to be aligned with the mainstream methodologies of frailty assessment within Oncology and other medical specialties—an initial frailty screening test to identify patients at high risk of poor clinical outcomes, who may benefit from structured and regularly performed CGA [58]. The identification of an individual with high severity of frailty could trigger protocols for even closer monitoring of adverse effects of treatment such as infection, nephrotoxicity, hypertension, and other cardiovascular and kidney complications. Follow-up frailty screening and subsequent CGAs could then be conducted to determine the need for a shift to a less-aggressive approach, change in treatment strategy, or, if required, redirection towards palliative care [89]. As described further in this section, the approach to assessing and responding to frailty may differ slightly with localized kidney cancer compared with metastatic disease and compared to settings where patients are complex [89].

In localized kidney cancer, frailty screening and subsequent CGA inform patients and clinicians whether active treatment, active surveillance, or watchful waiting may be the appropriate management option, considering surgical risks, treatment morbidity, and the competing risk of cancer-specific mortality [89]. For example, pre-operative surgical risk assessment in RCC utilizes the physical status score of the American Society of Anesthesiologists (ASA), a composite score to categorize patient function based on their pre-operative health status [90]. The major risks elicited from this scoring are potential cardiac or respiratory issues during anesthesia and mechanical ventilation. An ASA score ≥ 3 should alarm the practitioner to reconsider the benefits expected from surgical intervention in light of these risks. The ASA has often been combined with frailty scoring to guide clinical decision-making in surgical oncology [75,91,92]. Frailty screening tools that have been validated for use in onconephrology include simple questionnaires such as the G8 screening tool, which discriminates between older patients with increased risk of geriatric syndrome deficiencies and those without [93]. Seven questions (loss of appetite, loss of weight, mobility, neuropsychological problems, body mass index, polypharmacy, and perceived health condition) and the patient’s age in the G8 screening tool give 0, 1, 2, or 3 points each, with the addition cumulating to a total score. Individuals with a score of fewer than 14 points should be referred to a gerontologist or a specialist in geriatric oncology, where the CGA and its processes will be commenced. Other frailty screening tools validated for use in onconephrology include the Triage Risk Screening Tool (TRST) and the Vulnerable Elders-13 Survey (VES-13) [94,95]. Screening for physical frailty using the FFP has been utilized as well [96,97].

Where there is metastatic kidney cancer, choosing an appropriate frailty assessment method is important in providing guidance for prognostication, adjusting to systemic treatment regimens to limit treatment-related toxicities, or even avoiding treatment altogether [98]. There remains a paucity of data surrounding the best frailty assessment tools to use to guide clinical decisions for systemic treatment. Nevertheless, several scoring systems integrating components of frailty assessment and cancer-related prognostic factors have been developed. The most prominent scores applied within this context include adapted versions of the Memorial Sloan Kettering Cancer Center (MSKCC) score and the International Metastatic RCC Database Consortium (IMDC) [99,100,101,102]. The MSKCC is derived from a 2002 retrospective study of metastatic RCC patients formerly treated with interferon and is composed of five equally weighted criteria: two clinical criteria [<1 year from the time of diagnosis to systemic therapy and Karnofsky performance index status (KPS) < 80% (where ‘100%’ is the maximal score representing ‘perfect’ health and ‘0%’ represents death)] and three biological criteria (lactate dehydrogenase and corrected calcium over the upper limit and haemoglobin under the lower limit) [99]. MSKCC scoring classifies individuals into ‘good’, ‘intermediate’, and ‘high’ risk groups. For reference, the estimated median survival for these three groups was 20, 10, and 4 months, respectively, during the pre-targeted therapy era. Previous observational studies applying MSKCC scoring in older metastatic RCC populations have highlighted the good prognostic accuracy of the score in identifying clinical outcomes and treatment complications following VEGF tyrosine kinase inhibitor initiation [103,104]. The IMDC classification was initially derived from a 2009 retrospective study conducted for metastatic RCC patients treated with VEGF tyrosine kinase inhibitors [101,102]. It relies on the same two clinical and four biological criteria (platelet count, neutrophil count, corrected calcium over upper the limit, and haemoglobin under the lower limit) which are related to overall survival, and the score categorizes individuals into three prognostic groups: ‘favorable’ (0 criteria), ‘intermediate’ (1 or 2), and ‘poor’ (3 or more). The estimated median survival for each of the three groups is 43.2, 22.5, and 7.8 months, respectively. The IMDC classification has also subsequently been validated in metastatic RCC patients treated with immuno-oncology agents [105]. The latest European Association of Urology guideline advocates the usage of the IMDC classification to guide first-line systemic therapy prescription in metastatic RCC for older patients [106]. It suggests using KPS as a surrogate for frailty assessment within this setting, where KPS > 80% is the threshold when considering a patient to be frail.

Modifiable risk factors of poor outcomes in cancer, such as hypertension, obesity, functional limitation, poor nutritional intake, smoking, diabetes mellitus, alcohol consumption, and regular non-steroidal anti-inflammatory drug use should be considered together with findings from frailty screening and CGA to provide an accurate balance of risks versus benefits of treatment in patients with localized kidney cancer, metastatic disease, and, more importantly, those with cancer and co-morbidities including CKD. The age-adjusted version of Charlson’s co-morbidity index has been proposed as a valuable tool in this setting to prognosticate cancer-specific, treatment-specific, and overall clinical outcomes [107].

## 5. Recommendations for a Summarized Frailty Assessment Tool in Onconephrology

The sections above highlighted various frailty assessment tools that have been applied in nephrology, oncology, and onconephrology as well as in geriatric medicine in general. Frailty is a multi-dimensional syndrome. Whilst each frailty assessment tool may assess some aspects of frailty, there is no single tool that can assess total frailty. The assessment of frailty across the various tools is deemed to be inconsistent. A systematic review evaluating frailty screening methods for predicting the outcome of a CGA in older patients with cancer has shown weak to moderate agreement between performance status, FFP, Geriatric 8 (G8), and FI scores [108]. These findings indicate multiple frailty assessment tools need to be used for the holistic evaluation of frailty status. Though FFP, G8, and FI are relatively simple tools to use; conducting multiple assessments in itself is a time-consuming task that may not be ideal within a busy clinical environment. This suggests the need for a summarized frailty assessment method for clinicians.

Within the context of onconephrology, there have been recommendations to utilize a ‘simple value score’ based on age to determine the extent of frailty. A recently published study by Togashi and colleagues [109] investigated frailty-based biological ages using frailty-discriminant scores (FDS) and examined the effect of biological–expected life age discrepancy on the prognosis of patients with urological cancers. The FDS is a comprehensive frailty assessment tool including eGFR as a frailty parameter [110]. There are few frailty assessment tools that currently include kidney function as a parameter, and this was addressed in the FDS. Togashi and colleagues retrospectively evaluated frailty in 1035 patients diagnosed with urological cancers. Frailty-based biological age was defined by the FDS, using 1790 non-cancer individuals as controls. An expected life age (i.e., chronological age + life expectancy) was then subsequently calculated using the 2019 life expectancy table. This study included 405, 466, and 164 patients diagnosed with prostate cancer, urothelial carcinoma, and renal cell carcinoma (RCC), in which the median chronological age, life expectancy, and estimated frailty-based biological age were 71, 17, and 83 years, respectively. The investigators noted biological–expected life age discrepancies in urological cancers overall, localized diseases specifically, and metastatic diseases specifically were −4.8, −6.3, and +0.15 years, where a biological–expected life age discrepancy of >5 years was significantly associated with poor overall survival. Therefore, it was suggested that biological–expected life age discrepancies between frailty-based biological age and expected life age may be helpful in understanding the role of frailty in this setting.

Based on preliminary evidence, the concept of translating frailty status into a simple, summarized value such as age is certainly promising. It reduces the complexity and confusion of interpreting different scores/cutoffs from multiple frailty assessment tools, as well as saving time and effort for clinicians when conducting frailty assessments. Nevertheless, further studies are required to confirm the clinical validity of the summarized frailty assessment approach.

## 6. Summary and Future Areas of Development for Frailty Assessment in Onconephrology

**Figure 1:**
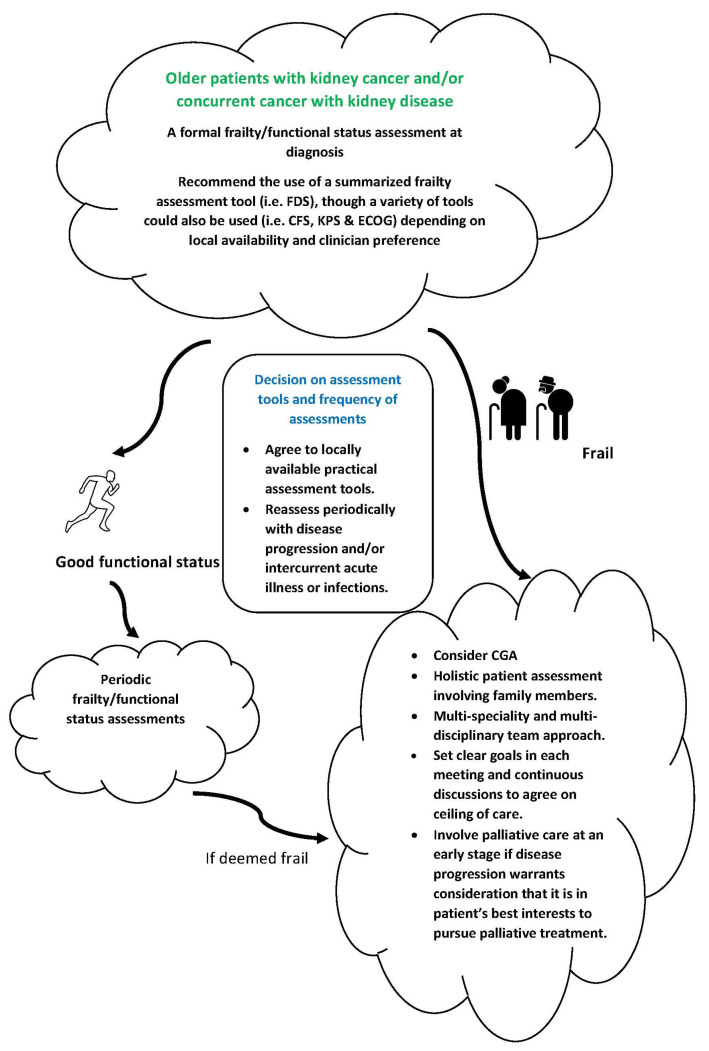
Proposed approach to frailty and functional status assessments in older patients with kidney cancer and/or concurrent cancer with kidney disease. CGA: comprehensive geriatric Assessment; CFS: clinical frailty scale; FDS: frailty-discriminant scores; KPS: Karnofsky’s performance status; ECOG: Eastern Cooperative Oncology Group score.

We have seen a greater awareness of the concepts surrounding frailty and frailty assessment, and its implications in the medical field including onconephrology over recent years. It is encouraging that the approaches to assess frailty status and associated geriatric syndromes specific to onconephrology are continuously expanding. This is a complex task; however, there are uncertainties in regard to frailty assessment within Onconephrology that require further exploration. Relatively established and commonly used frailty measures (i.e., the measures highlighted in the first section of our paper) have not yet been applied and validated for use in onconephrology. More work to determine whether and which of these tools are useful within the onconephrology context is needed, whilst the development of a simplified and summarized frailty assessment tool optimized for clinical practice needs to be continuously explored. There should be greater involvement of other allied health professionals, such as primary care clinicians and pharmacists, during the process of frailty assessment given their roles in monitoring and managing the patient’s response to systemic treatment. Considering the unique challenges and multi-faceted complications of onconephrology presentations, the development of a more onconephrology-specific CGA model would be desirable. Ultimately, an onconephrology-specific CGA model should provide greater emphasis towards patient values and goals of care, namely that many older, frailer cancer patients may be less likely to trade QoL for an improvement in overall survival compared to younger, fitter patients. This may be more apparent if they are living with significant frailty severity and a poor outlook with respect to survival. Considering the existing evidence, Figure 1 illustrates our proposed approach to the periodic assessment of frailty and its associated geriatric impairments in onconephrology, with the awareness that frailty is dynamic over time and frailty status trajectories vary for each individual [111]. We acknowledge further research to validate that our advocated approach is required. Going forwards, we anticipate greater efforts to address current knowledge gaps and initiatives to integrate frailty assessment in onconephrology with hopes to identify and optimize personalized, goal-directed management strategies for this patient population.

## Figures and Tables

**Table 1:** Characteristics of commonly used frailty measures in studies of kidney disease and oncology.

Factor	Fried Frailty Phenotype	Frailty Index	Tool Based on Clinical Impression (i.e., Karnofsky Performance Index, Eastern Cooperative Oncology Group Performance Status, and Clinical Frailty Scale Score	Objective Measurement Tool (i.e., Short Physical Performance Battery)
Type of Measure	Binary (frail or not)	Continuous	Ordinal	Binary or Ordinal
Data Source	Physical exam/questionnaire	Multiple including administrative data	Clinical judgment (provider or patient)	Physical examination
Ease of Capture	Mixed, depending on the traditional or modified version	Mixed, depending on the availability of information sources or the need for primary capture	Very easy/point of care	Mixed (may require face-to-face assessment and equipment)
Ease of Understanding (Patients and Providers)	Very easy	Potentially difficult	Very easy	Very easy
Construct Validity	Low	High	Moderate	Mixed (depending on how used)
